# Stress and Pain. Predictive (Neuro)Pattern Identification for Chronic Back Pain: A Longitudinal Observational Study

**DOI:** 10.3389/fmed.2022.828954

**Published:** 2022-05-10

**Authors:** Pia-Maria Wippert, Laura Puerto Valencia, David Drießlein

**Affiliations:** ^1^Medical Sociology and Psychobiology, University of Potsdam, Potsdam, Germany; ^2^Faculty of Health Sciences, Joint Faculty of the University of Potsdam, Brandenburg Medical School Theodor Fontane, and the Brandenburg University of Technology Cottbus-Senftenberg, Postdam, Germany; ^3^Statistical Consulting Unit StaBLab, Ludwig-Maximilians-Universität München, Munich, Germany

**Keywords:** allostatic load index, hair cortisol, low back pain, psychosocial moderators, hypocortisolemic symptom triad, stress types

## Abstract

**Introduction:**

Low back pain (LBP) leads to considerable impairment of quality of life worldwide and is often accompanied by psychosomatic symptoms.

**Objectives:**

First, to assess the association between stress and chronic low back pain (CLBP) and its simultaneous appearance with fatigue and depression as a symptom triad. Second, to identify the most predictive stress-related pattern set for CLBP for a 1-year diagnosis.

**Methods:**

In a 1-year observational study with four measurement points, a total of 140 volunteers (aged 18–45 years with intermittent pain) were recruited. The primary outcomes were pain [characteristic pain intensity (CPI), subjective pain disability (DISS)], fatigue, and depressive mood. Stress was assessed as chronic stress, perceived stress, effort reward imbalance, life events, and physiological markers [allostatic load index (ALI), hair cortisol concentration (HCC)]. Multiple linear regression models and selection procedures for model shrinkage and variable selection (least absolute shrinkage and selection operator) were applied. Prediction accuracy was calculated by root mean squared error (RMSE) and receiver-operating characteristic curves.

**Results:**

There were 110 participants completed the baseline assessments (28.2 ± 7.5 years, 38.1% female), including HCC, and a further of 46 participants agreed to ALI laboratory measurements. Different stress types were associated with LBP, CLBP, fatigue, and depressive mood and its joint occurrence as a symptom triad at baseline; mainly social-related stress types were of relevance. Work-related stress, such as “excessive demands at work”[*b* = 0.51 (95%CI -0.23, 1.25), *p* = 0.18] played a role for upcoming chronic pain disability. “Social overload” [*b* = 0.45 (95%CI -0.06, 0.96), *p* = 0.080] and “over-commitment at work” [*b* = 0.28 (95%CI -0.39, 0.95), *p* = 0.42] were associated with an upcoming depressive mood within 1-year. Finally, seven psychometric (CPI: RMSE = 12.63; DISS: RMSE = 9.81) and five biomarkers (CPI: RMSE = 12.21; DISS: RMSE = 8.94) could be derived as the most predictive pattern set for a 1-year prediction of CLBP. The biomarker set showed an apparent area under the curve of 0.88 for CPI and 0.99 for DISS.

**Conclusion:**

Stress disrupts allostasis and favors the development of chronic pain, fatigue, and depression and the emergence of a “hypocortisolemic symptom triad,” whereby the social-related stressors play a significant role. For translational medicine, a predictive pattern set could be derived which enables to diagnose the individuals at higher risk for the upcoming pain disorders and can be used in practice.

## Introduction

Low back pain (LBP) is the leading cause of disability worldwide. In 2015, approximately 540 million individuals were affected by activity-limiting LBP one time during the year (1). Although the prevalence rates are high, most individuals express no pathological causes and recover quickly, but around 8.5% develop non-specific persistent pain and disability (2). There is evidence that a range of biological, psychological, and social factors contribute to the development of chronic low back pain (CLBP) that accompany impaired function in daily life, reduced social participation, and financial welfare (3).

In particular, stress is discussed as an important risk factor for the development of non-specific CLBP within the yellow flag concept (4). Stress can be both a trigger or/and an amplifier of pain. For example, early life trauma [e.g., pain prone patients (5)] or the accumulation of adverse life events (6) have been described as triggers of pain. In fibromyalgia, stress was an amplifier for pain (7, 8). These two effects may be based on neuroendocrine and psychophysical responses during stress experience, influencing pain perception and pain processing by multiple neuro–functional processes.

During the stress response, neurotransmitters and hormones are released. These processes take place in the so-called stress triangle, which comprises the ergotropic (noradrenergic bundle: Locus coeruleus/sympathetic nervous system, or working system), the glandotropic system (paraventricular nucleus, pituitary gland, adrenal glands, glucocorticoid receptors, or energy supply system), and the trophotropic [raphe nuclei/parasympathetic nervous system, or recovery system (9, 10)]. In this triangle, allostasis and the adaption to stress are organized. During prolonged stress, the glandotropic system habituates, while the ergotropic system remains overactive. This asynchronous change of the involved systems disturbs the body’s own tuned protective mechanism of allostasis and leads to an accumulation of physiological imbalances in the long-term, the so called allostatic load (11, 12). One example for such an imbalance regarding the association between stress and pain disorders is the reduced hypothalamus–pituitary–adrenal axis (HPA) activity due to cortisol deficiency (7, 8). This so-called “hypocortisolism symptom triad” comprises the joint occurrence of pain, depression, and fatigue as a result of chronic stress.

The interface between stress and pain is complex because of the various physiological pathways by which pain disorders could be triggered or amplified. First, the messenger substances released during stress exposure [e.g., neurochemical transmitters such as norepinephrine, acetylcholine, dopamine, cytokines, neuropeptides, glutamate, gamma-aminobutyric acid (GABA)] can influence nociception at the peripheral level (recruitment and sensitization) as well as nociceptive processing at the spinal level (signal cascades, afference, and efference) (13). These same transmitters play a further role in the modulation of the descending serotonergic and noradrenergic signals from the brainstem influencing the central reciprocal pain inhibition (14). Additionally, these chemical alterations reduce the amount of nerve growth factor which plays an important role in the differentiation of Aδ or C-fibers, their innervation density and therefore their transmission quality. Second, the stress response is controlled by a synaptic information from the various brain regions, such as the limbic system (including the hippocampus and amygdala) or the brain stem, all involved in the processing of pain stimuli (areas of the pain matrix). Prolonged stress exposure leads to altered connectivity in the pain matrix, to a reduction in cell proliferation and gray matter volume, and to a reorganization of the brain areas of the pain matrix (11, 15). Third, stress-related changes in the metabolic system (e.g., local fat depots, cholesterol in plasma membranes) can influence myelination, peripheral nerve functions (16) and pain transmission (17).

Accordingly, stress is associated with pain and is an important factor in developing chronic pain. However, which types of stress are most relevant and which underlying mechanisms is still not fully assessed. A more differentiated comprehension of such stress-related mechanisms on musculoskeletal problems and pain (15) would be necessary for the development of more concrete therapeutic treatments and diagnostics (12). Until now, mostly multimodal treatments are generic, and they are not considering specific personal needs. For this reason, a simultaneous assessment of different psychobiological interactions within the stress triangle would be beneficial. It would allow an identification of important predictive stress patterns in the development of chronic pain. In this regard, a predictive pattern set could be the basis for the derivation of a diagnostic tool, as it was done for burnout syndrome (10, 18). Here, a specific Neuropattern (10) diagnostic for burnout symptoms was developed, which is unfortunately still missing with regards to the non-specific pain syndromes (19). Therefore, this study aims to the following factors:

(1) Analyze the associations between different types of stress with non-specific current LBP and its influence on the development of non-specific CLBP as well as on fatigue and depressive mood (as individual outcomes or as symptom triad) within 1 year.

(2) Identify the most important stress types regarding the development of non-specific chronic LBP, fatigue, and depressive mood within 1 year.

(3) Identify the most predictive stress-related (neuro) pattern set regarding the development of non-specific CLBP within 1 year and to test its accuracy for diagnostics.

## Materials and Methods

### Study Design

This observational longitudinal study includes four measurement time points (M1–M4), every 4 months, for a total duration of 1 year. The measurements consisted of hair samples and standardized questionnaires at each time point (M1–M4), as well as blood, urine samples together with clinical and laboratory parameters collected only at baseline (M1) and at the 1 year follow-up (M4) by medical nurses. The study was conducted between August 2013 and June 2015.

### Participants

The individuals who were seeking back pain treatment at the Ernst von Bergmann clinic and the outpatient clinic of the University of Potsdam were recruited through announcement at the University Potsdam. In total, 140 subjects with intermittent non-specific LBP between 18 and 45 years of age took part in the study. The participation was not compensated, but the participants received their examination data and an individual stress profile after study completion. Convenience sampling technique was used.

Inclusion criteria were listed as follows: At least one episode (≥4 days) of non-specific LBP in the last 12 months [according to the national treatment guideline NVL (20) and ICD-10: M50-54; LBP appearance defined as a minimum pain intensity score of 20 on a pain 100-point visual analog scale (VAS)], ability to understand the content of the study and to fill in a German questionnaire independently. Exclusion criteria were acute infections, pregnancy, hormonotherapy or the intake of certain types of medication (e.g., antibiotics and glucocorticoids), particular diseases (e.g., cardiovascular, metabolic diseases, thyroid disorders, vascular, malign, lung, liver or autoimmune diseases, hemophilia or psychological disorders, e.g., ICD-10: F70-79), inability to fill in a questionnaire and hair shorter than 2 cm. All participants signed a written informed consent after receiving written and oral information about the study by a study nurse.

The defined criteria on maximal age (individuals aged less than 45 years) was based on epidemiological studies, which indicated that the chronic courses after an acute LBP episode increased abruptly from 40 years of age. Therefore, with a preventive perspective, risk patterns for developing CLBP should be identified earlier (2).

**FIGURE 1:**
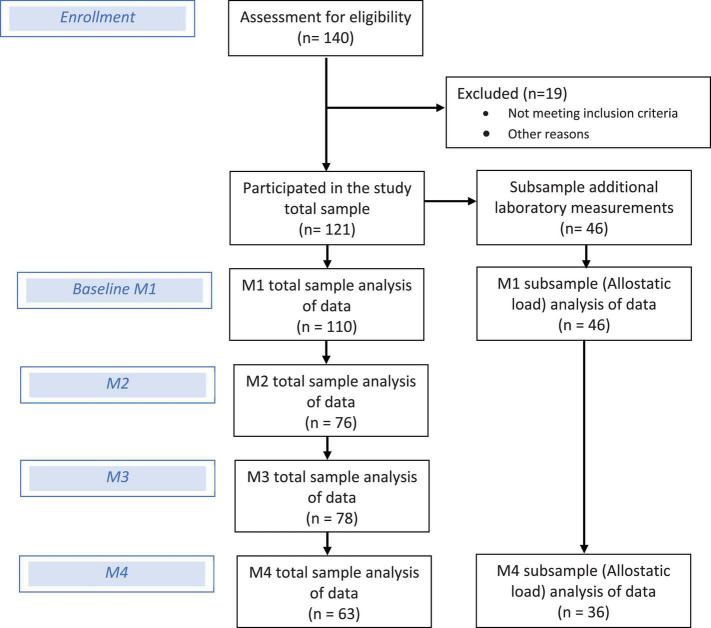
Flow diagram of participants and sample analyzed data.

Further, 46 individuals agreed and committed to the protocol of an additional comprehensive medical and laboratory test battery (see Figure 1). This protocol included avoiding certain foods (coffee, tea, alcohol, bananas, cheese, nuts, vanilla, and citrus fruits), intensive physical activity (>2 h/day) and medication, as well as collecting one’s own urine from 7 p.m. to 7 a.m. (12 h) 1 day before the examination. The fasting blood tests (12 h food abstinence before blood withdrawal) and the medical examination took place between 7 a.m. and 8:00 a.m. at the University of Potsdam outpatient clinic. The blood samples were evaluated both at the outpatient clinic and clinic laboratory of the University of Potsdam, while the hair samples were analyzed in the laboratory of biopsychology of the Technical University of Dresden.

The sample size calculation for the minimal number of subjects required to detect an association between psychometric stress measures and pain disability was based on a medium effect size *f*^2^, as well an α*-*error probability of 5%, a β-error probability of 20%, suggesting a *n* = 85 [power analysis by G*Power (21)]. The sample size calculation for detecting differences between stress physiological measures [allostatic load index (ALI) scores] within 1 year was based on a medium Cohen’s *d* (0.5) effect size, as well an α-error probability of 5%, a β-error probability of 20%, suggesting a *n* = 34.

### Ethics Approval

All clinical investigations, and measures have been conducted according to the principles expressed in the Declaration of Helsinki. The final ethics approval was provided on 6 May 2013 by the major institutional ethics review board of the University of Potsdam, Germany (No. 44/2012).

### Assessments

**FIGURE 2:**
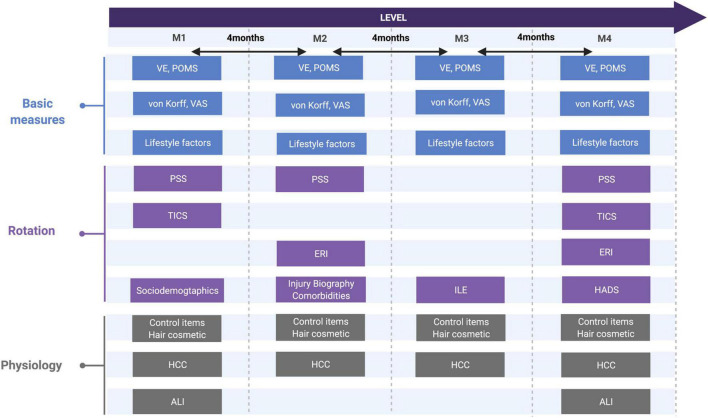
Study design regarding psychometric and biological measures and its rotation. von Korff, CPG, chronic pain grade questionnaire; VAS, visual analog scale; VE, vital exhaustion, TICS: trier inventory of chronic stress; PSS, perceived stress scale; ERI, effort-reward-imbalance; ILE, inventory of life-changing events; POMS, profile of mood status; HADS, hospital anxiety depression scale, sociodemographic and lifestyle factors, hair cosmetic, comorbidities, ALI, allostatic load index; HCC, hair cortisol concentration.

The outcomes non-specific current and chronic LBP, fatigue, and depression were assessed by the standardized questionnaires at each measurement point (M1–M4, Figure 2). The exposure or predictor criteria stress was operationalized using both psychometric and physiological/biometric data. Further, sociodemographic and lifestyle factors [alcohol (glasses per week, type of alcohol); tobacco consumption (cigarettes a day, pack years); medication (type, daily dosage); physical activity (frequency per week, duration of unit, intensity according to the WHO guidelines (22)); sleep (quality on a 0-10 Likert scale)] were documented for all participants at all time points.

### Outcomes

#### Chronic Pain

Chronic pain was assessed by the German version of the chronic pain grade questionnaire (23) [original English version (24)], which consisted of 7 items; 1 item considered the days of chronic pain and 6 items rated the chronic pain on an 11-point numeric rating scale). The original questionnaire showed a good internal consistency (Cronbach’s alpha of 0.91) and good correlations (*p* < 0.001) with the equivalent dimensions of the Short Form 36 Health Survey Questionnaire (convergent validity) cross-sectional (25) and over time in a general practice population in Scotland (26). The translated German version used in our study showed good internal consistency (Cronbach’s alpha reported was 0.82), it was significantly correlated with other clinical variables; moderate to high with instruments assessing patient’s disability, and weak to moderate but significant with grading and staging chronic pain measurements, all within a population of primary care back pain patients (23). The questionnaire operationalized the severity of pain syndromes on the following two subscales: Characteristic pain intensity (CPI; 0 = “no pain” to 100 = “the worst pain imaginable”) and the subjective pain disability (DISS; 0 = “no disability” to 100 = “I was incapable of doing anything”) within the past 3 months. Both subscales were defined as the mean of three individual numeric rating scales questions. The high quality of the questionnaire was also confirmed in the context of the International Classification of Functioning, Disability and Health (ICF) (27). Cronbach’s alpha in our sample was α = 0.92. Current pain intensity (acute pain) was assessed by a horizontally presented 100-mm VAS [0 = “least possible pain” and 100 = “worst possible pain” (28)].

#### Fatigue

Fatigue was evaluated by the short version of the Maastricht questionnaire vital exhaustion (VE) [9-Item version (29, 30)] and the fatigue subscale from the German short version Profile of Mood States Questionnaire [POMS (31, 32)]. The VE questionnaire included nine questions about disturbed sleep, extreme fatigue, mental and physical irritability, and feelings of hopelessness with four possible answers (“no” = 0; “undetermined” = 1; “yes” = 2). The scale total score ranges from 0 to 18 (scores up to 4 indicate mild-to-moderate exhaustion). Cronbach’s alpha in the sample was α = 0.71. The POMS fatigue subscale included 7 items to be ranked on a 7-point Likert scale ranging from 1 (= “not at all”) to 7 (= “very strong”), so the total score ranged from 0 to 42.

#### Depressive Mood

Depressive mood was assessed by one scale of the German short version of the POMS (31, 32). The depression subscale comprised 14 mood relevant adjectives that had to be ranked on a 7-point Likert scale ranging from 1 (= “not at all”) to 7 (= “very strong”). Subjects completing the POMS were asked to reflect on their emotional states over the past week. The internal consistency in this sample was at α = 0.88.

### Predictor Criteria/Exposure

Stress was defined though psychometric and physiological variables as discussed in the following sub-sections:

#### Stress Psychometric Tests

The extensive psychometric test battery to measure the different stress types led to the decision to offer questionnaires only in rotation, not at every measurement point as far as feasible in form and content (assessment time frames and stability of attributes). The aim was to record a broader spectrum of stress-related assessments while preserving the motivation of the participants. Long-term prediction should not be limited due to this rotation as assessments at M1 and M4 were complete.

The types of stress were assessed by the following questionnaires: The “Trier Inventory of Chronic Stress” [TICS (33)] with its 57 items (rated on a 5-point Likert scale, from 0 = “never” to 4 = “very often”) was used for the assessment of chronic stress in the past 3 months. The items are summed up to nine scales of potential chronic stress domains such as “work overload, social overload, pressure to perform, work discontent, excessive demands at work, lack of social recognition, social tensions, social isolation, and chronic worrying.” Cronbach’s alpha in the sample was α = 0.95.

Perceived stress was assessed by the Perceived Stress Scale (PSS) (34, 35), a questionnaire with 10 items asking about stressful situations during the last 3 months (e.g., “In the past 3 months, how often have you been upset because of something that happened unexpectedly?”). The sum of the answers on a 5-point Likert scale (0 = never to 4 = “very often”) gave information about the corresponding stress value ranging from 0 (no perceived stress) to 40 (high perceived stress). Cronbach’s alpha was α = 0.86.

Stress at work was measured by the effort–reward–imbalance questionnaire (ERI) (36), which consisted of 16 items measuring effort, reward (general reward, esteem, job promotion, and job security),and over-commitment on a 4-point Likert scale (0 = “not true at all” to 3 = “completely true”). Furthermore, an additional scale sets effort and reward into relation. Cronbach’s alpha was α = 0.64.

The inventory of life-changing events counts for type, number and load of critical life events (ILE) (37), whereby participants rate 40 critical life-events regarding occurrence, frequency, and year of occurrence. For the analysis here, only the scale for the total number of life events was used, for which all critical life-events over lifespan were summed up ranging from 0 to 40.

Stress burden was operationalized by a total stress index which was created by using a median split (score below the median = 0 or above the median = 1) for each stress questionnaire (TICS, PSS, and ERI). Afterward, the zeros and ones were summed up to an individual stress index. As the total number of life events referred more to a lifespan perspective, the ILE was not sub-summarized in the total stress index, which included mainly tests covering the last months.

#### Stress Physiological Tests

Regarding the stress burden and the stress-related reactivity of different neurobiological interfaces, mostly analytical techniques and methods less susceptible to daily fluctuations were chosen (38). Also, ALI and hair cortisol concentration (HCC) were not dependent on daily fluctuations and cycle phases and were used in this study. The validity of the methods is well confirmed in different previous studies (39, 40). Within an additional pilot study, HCC and ALI showed a high test-retest reliability of the biomarkers within a 24-h time frame (38).

Allostatic load index represents the physiological load accumulated in the body through prolonged physical or mental stress (11, 12). Current standards suggest an evaluation of 24 indicators on different levels/systems to define total ALI (39). These indicators include the sympathetic nervous system (12-h urinary adrenaline and noradrenaline), parasympathetic nervous system (four heart rate variability indicators measured through electrocardiogram), HPA axis [12-h urinary cortisol and serum dehydroepiandrosterone sulfate (DHEA-S)], immune system [C-reactive protein and fibrinogen in plasma, interleukin-6, E-selectin, and intercellular adhesion molecule 1 (ICAM-1) in serum], cardiovascular system [systolic blood pressure (BPSYS) and diastolic blood pressure (BPDIA), resting heart rate], fat metabolism [body mass index, waist-hip ratio, triglycerides, high density lipoprotein (HDL) cholesterol, and low density lipoprotein (LDL) cholesterol] and sugar metabolism (glycohaemoglobin, fasting glucose and insulin) (41). The biomarkers assessed are discussed as in the following:

**(1) Sympathetic nervous biomarkers:** Analysis of urinary epinephrine and norepinephrine levels from 12-h overnight urine collections were performed *via* ELISA (norepinephrine RE5926, epinephrine RE59251, both ILB International GmbH Germany).

**(2) Parasympathetic nervous biomarkers:** Electrocardio graphic (Holter ECG, Schiller MT-101) electrodes were placed in the left lower quadrant on both shoulders. The ECG activity was monitored over an 11-min seated baseline period to assess heart rate variability indicators (SDNN, rMSSD, SDANN, SDNNidx) and resting pulse. The ECG was additionally standardized by breathing rhythm.

**(3) The HPA biomarkers:** Urine cortisol (μg/day) was measured with ELISA (RE52241, ILB International GmbH Germany). The DHEA-S was measured with ELISA (RE52181, TECAN Hydro Flex, ILB International GmbH Germany).

**(4) Immune system biomarkers:** Soluble E-selectin (sE-selectin), soluble ICAM-1 as well as interleukin-6 were assessed with enzyme-linked immunosorbent assays (BE59011 for sICAM-1, BE59061 for sE-selectin and BE58061 for IL-6; all from IBL International GmbH, Hamburg Germany). Further, C-reactive protein was measured by an immunoturbidimetry latex test (ABX Pentra 400) and Fibrinogen by traditional turbidimetry according to Claus (Siemens BCS XP).

**(5) Cardiovascular biomarkers:** Outpatient nurses assessed BPSYS and BPDIA 3 times, each separated by a 30-s rest period. The final blood pressure scores were obtained by averaging the values of the second and third measurements (BOSO BS 90 Blood pressure instrument, BOSCH + SOHN GmbH u. Co., KG, Jungingen, Germany).

**(6) Lipid metabolic biomarkers:** Triglycerides, HDL cholesterol and LDL cholesterol were assessed *via* enzymatic colorimetric assays (ABBOTT Architect ci8200; Abbott Laboratories, IL, United States). Furthermore, weight (Kern MPS scale; Kern & Sohn GmbH, Balingen, Germany), height (Seca 222 telescopic measuring rod; seca ag, Suisse) and waist/hip circumference (customary measuring tape) were measured, whereby hip circumference was assessed above the umbilicus at the narrowest point between ribs and the iliac crest, the hip circumference at the widest point across the buttocks. Body mass index (BMI) was calculated as weight in kg/(height in m)^2^.

**(7) Glucose metabolic biomarkers:** Glycosylated hemoglobin (HbA1c) was analyzed with HPLC Bio–Rad Variant II (Bio–Rad Laboratories, CA, United States), fasting glucose *via* a hexokinase enzymatic reaction using the Roche Cobas 400 plus (Roche Diagnostics Ltd., Basel, Switzerland), and fasting insulin by an electrochemiluminescence enzyme immunoassay (ECLIA), using Roche Cobas 8000 Modul E620 (Roche Diagnostics Ltd., Basel, Switzerland). Insulin resistance [*via* Homeostasis model assessment index (HOMA)] was calculated using the formula: glucose [mg/dl] × insulin [μU/ml]/405).

The ALI was calculated by classifying each biomarker into quartiles within the sample: subjects with values in the fourth quartile (>75%) were assigned the value 1 (loaded), while the rest were assigned the value 0 (unloaded). Subsequently, ALI was created by summarizing the values for each test person across the 24 biomarkers [ALI-total (39, 40)]. Furthermore, stress burden was represented more differentiated by ALI-sub-scores as discussed in the following: The ALI-I (ALI-primary) represents mediators of the primary stress response and physiological adaptation (first defense line including cortisol, noradrenalin, adrenalin, and DHEA-S). The ALI-II (ALI-secondary) expresses secondary mediators which are involved in the prolonged maladaptation to chronic stress (HbA1c, ratio of total cholesterol to HDL, LDL, BPSYS, BPDIA, and waist–hip ratio (WHR) (40).

Hair cortisol concentration was extracted from two thin hair strands taken from the back of the head below the covering hair (diameter approximately 2 mm; length, 2 cm) and analyzed by ELISA, IBL RE62019 (42). One centimeter of hair corresponds to a 1-month measurement period, so a total of 4 months measurement period was covered retrospectively.

### Statistical Analysis

Psychometric tests were prepared in line with test manuals. Physiological data were controlled for outliers and treated along analysis kit recommendations. The total stress burden was represented by aggregated variables to map a subjective accumulation of stress (total stress index) and a biological accumulation of stress (ALI).

Descriptive and inferential statistics were applied to the data using SPSS (IBM 24.0) and R (43). Regarding the first study objective, the multiple linear regression models for main effects (M1 and M4) were used, whereby these calculations were controlled for age, sex, and the baseline value of the respective variable. For the second study objective, identification of the most predictive stress type concerning the symptom triad (pain, fatigue, and depressive mood), selection procedures for model shrinkage and variable selection [least absolute shrinkage and selection operator (LASSO) (44)] were performed. Considering the third study objective, LASSO was applied on a variable basis for selection of the best predictor set (once for biomarkers, once for psychometric items and then sub-summarized to a psycho and bio-set). Only metric indices were used for the LASSO analysis. Afterward, root mean squared error (RMSE) for prediction accuracy of each set, was calculated for pain intensity and disability. Finally, receiver-operating characteristic curve (ROC) and area under the curve (AUC) were performed to assess the model’s ability to discriminate (only applied to the selected biomarker model). The dichotomous reference needed to compute ROC curves classifying low and high risk patients was defined as equal or more than 30% (45) of pain intensity and disability (corresponding to 30 points of the 100 on the von Korff scales). Further, the AUC corrected for optimism using bootstrapping (1,000 iterations) was reported. All LASSO models and RMSE calculations were controlled for lifestyle factors that had a significant effect on the outcomes during previously performed LASSO selections [age, sex, tobacco, alcohol consumption, sleep, sports activity (logarithmic sports variable), monthly income as well as baseline pain/fatigue or depressive mood].

## Results

### Descriptive

A total of *n* = 121 participants took part in the longitudinal study, of which *n* = 110 were included in the analysis at baseline (age mean = 28.2 ± 7.5 years, 38.1% female, BMI mean = 23.4 ± 3.5 and WHR mean = 0.8 ± 0.7). On average, the subjects participated in physical activity (sports) 5.7 ± 5.1 h per week, 23.7% were academic professionals, 28.8% clerical support or sales workers, 10.9% craft and trades workers, 22% of the others included students. 35% reported a monthly net income under €1,250, 22.6% from €1,750–2,249, 17% from €1,250–1,749, and 24.5% over €2,250 (*n* = 53). The sample shows, on average, low to moderate values for chronic and acute pain complaints; at baseline CPI mean of 26.4 ± 18.3, DISS 12.2 ± 17.4, and current pain VAS 11.1 ± 16.8. Further, participants reported a moderate impairment due to fatigue and symptoms of depressive mood (see Supplementary Table 1). Physiological parameters were distributed within normal ranges. Allostatic load index was on average five with a maximum of 11 (possible range: 0–24, see Supplementary Figure).

### Stress Types and Burden in the Prediction of Pain, Fatigue, and Depressive Mood (Objective 1)

In this objective, the influence of stress types on current and on the development of non-specific back pain, fatigue, and depressive mood in individual appearance or as symptom triad was investigated.

#### Stress and Back Pain

Cross-sectional results (baseline M1) show that social and work-related stress types such as “Social overload, Social tensions, Excessive demands at work, or Work overload (from TICS), Perceived stress (from PSS),” and total stress index were significantly associated with current LBP pain intensity. Regarding CLBP “Excessive demands at work, Social tensions, and Perceived stress” were significantly associated with CLBP intensity, while “Social tensions, Perceived stress, Over-commitment (from ERI)” and critical life events were associated with CLBP disability. Considering the 1-year prediction of the pain development (M1 to M4 longitudinal), the results differ considerably; CLBP intensity is predicted by Chronic Worrying and current LBP intensity by critical life events and HCC (see Table 1).

**TABLE 1:** Main effects: (regression coefficients ß) for the influence of types and burden of stress (psychometric and physiological measures) on the outcome criteria back pain, mood, and fatigue as well as symptom triad (gray marked).

	Back pain						Fatigue				Depressive mood	

	Disability^1^		Intensity^1^		VAS		Fatigue VE^2^		Fatigue^3^		Depression^2^	
	**M1**	**M4**	**M1**	**M4**	**M1**	**M4**	**M1**	**M4**	**M1**	**M4**	**M1**	**M4**
**Chronic stress**												
Work overload^4^	0.45	−0.09	0.43	−0.13	0.54*	−0.52	0.25**	−0.01	0.48**	0.19	0.36**	0.26
Social overload^4^	0.62^#^	0.09	0.52	0.22	0.81*	−0.22	0.11	0.11	0.70**	0.50^#^	0.57**	0.39
Pressure to perform^4^	−0.06	−0.13	−0.03	−0.05	0.23	−0.21	0.16	0.16	0.59**	0.44*	0.29*	0.32
Work discontent^4^	−0.47	0.42	−0.11	0.57	−0.15	−0.10	0.18*	0.28*	0.23	0.06	0.35*	−0.06
Excessive demands at work^4^	0.74	0.23	0.93*	0.60	0.95*	−0.06	0.40**	−0.14	0.73**	−0.21	0.71**	−0.33
Lack of social recognition^4^	0.74	0.18	0.57	1.10^#^	0.19	0.15	0.30*	0.06	1.06**	0.15	0.99**	−0.54
Social tensions^4^	1.38*	0.38	1.08*	0.58	1.09*	0.68	0.16	0.26	0.96**	0.04	0.63**	0.56
Social isolation^4^	−0.26	−0.08	−0.15	0.33	0.20	−0.54	0.23*	−0.03	0.31	−0.44	0.63**	−0.60^#^
Chronic worrying^4^	0.52	1.23^#^	0.88^#^	1.36**	0.81^#^	1.12	0.68**	0.19	0.81**	0.03	0.91**	0.07

**Perceived stress** ^5^	0.82**	0.65	0.80**	0.56^#^	0.59*	0.60	0.37**	0.06	0.58**	0.07	0.62**	0.36

**Stress at work**												
Effort^6^	2.01^#^	0.97	1.13	1.05	1.70	0.76	0.50*	0.33	0.85^#^	1.53**	0.24	1.32*
Reward^6^	−0.46	−0.34	−0.20	−0.83	−0.59	0.20	−0.15	−0.04	−0.19	−0.45	−0.28	−0.16
Over-commitment^6^	1.17*	0.10	0.54	−0.08	0.50	−0.57	0.37**	0.11	0.57*	0.78*	0.18	0.80*

**Critical life events** ^7^	**0.96** *	−0.16	0.59	0.13	0.43	1.20**	0.05	0.19^#^	0.25	0.32^#^	−0.05	0.38^#^

**Stress burden**												
Total stress index	0.69	−0.43	0.28	0.12	1.51*	−1.73	0.55**	0.38	1.32**	0.65	0.72*	0.28
Total allostatic load	0.65	−0.28	1.25	−0.31	0.74	0.76	0.03	0.24	−0.03	0.66^#^	−0.44	0.03
Primary allostatic load	−0.62	−1.81	0.39	−1.92	1.03	3.02	−0.31	0.42	−1.04	−0.04	−1.14	1.17
Secondary allostatic load	−0.19	1.63	1.59	1.20	0.71	1.70	0.11	0.35	−0.47	2.05*	−1.20	1.06
HCC	−0.03	−0.12	−0.13	−0.10	0.10	−0.38*	0.04	−0.01	0.01	0.09	0.04	0.00

^#^p < 0.10, *p < 0.05, **p < 0.01, Bold values: p < 0.05, Linear Regression Models; adjusted by age, sex and baseline outcome (M1) in the case of M4. ^1^Chronic pain grade questionnaire (DISS, CPI); ^2^VE, vitale exhaustion; ^3^POMS, profile of moods questionnaire; ^ 4^TICS, trier inventory of chronic stress; ^5^PSS, perceived stress scale; ^6^ERI, effort-reward-imbalance-questionnaire, ^7^ILE, the inventory of life-changing events.

#### Stress and Fatigue

Chronic stress (all TICS scales), perceived stress, stress at work (Effort, Over-commitment) and total stress index showed associations to the current fatigue state measured in two different dimensions (VE and POMS, see Table 1). Regarding a 1-year prediction, work-related stress types such as “Pressure to perform, Work discontent, Effort, and Over-commitment” as well as physiological stress burden measured by the biomarker index allostatic load (ALI-secondary) had the strongest influence (see Table 1).

#### Stress and Depressive Mood

Cross-sectional results at baseline (M1) indicate that almost all chronic social stress types (TICS and PSS) and stress burden (total stress index) had an influence on the current mood. Moreover, these associations also referred to depressive mood in the future (upcoming 12 months) in the case of some work-related stress types (Over-commitment, Effort), although no biomarker association was found (see Table 1).

#### Stress and Symptom Triad

Including all *p*-levels (*p* < 0.01 up to *p* < 0.05), significant associations to the appearance of a symptom triad can be shown cross-sectionally for the stress burden (total stress index) and following stress types: “Social overload, Work overload, Excessive demands at work, Social tensions, and Perceived stress.” An upcoming symptom triad (longitudinal) is best described by an influence of Over-commitment (see Table 1 gray area).

**FIGURE 3:**
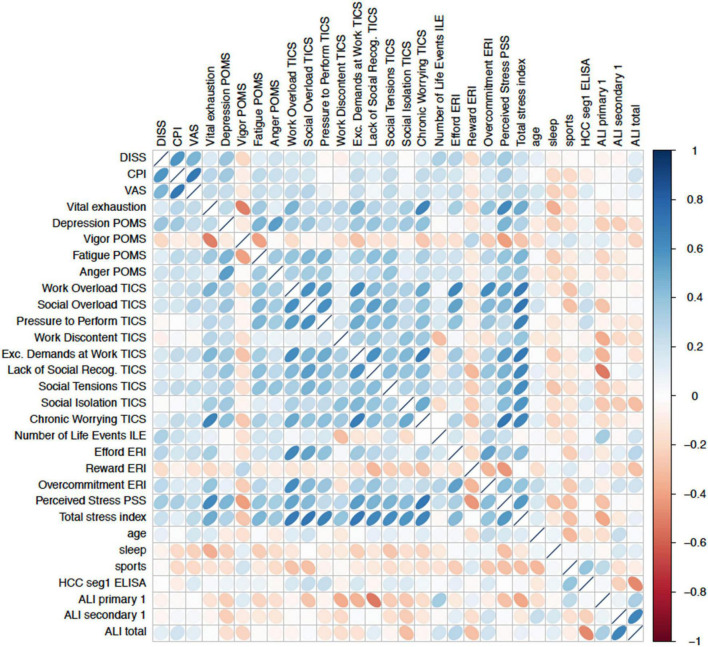
Correlation matrix of baseline measures demonstrate intercorrelations between psychometric stress scales, lifestyle factors (sleep, age, and physical activity) and the outcome criteria. Intercorrelations limit the significance of prediction methods such as linear multiple regressions models in identifying best predictors.

Inter-correlations regarding different stress types simultaneously within the multiple regression models are shown in Figure 3.

### Most Important Stress Types for the Development of Pain, Fatigue, and Depressive Mood (Objective 2)

Considering the stricter LASSO model for the 1-year prediction of non-specific CLBP, only “Excessive demands at work (from TICS)” out of all psychometric scales remained predictive for the development of disability (*b* = 0.51 [95%CI -0.23, 1.25], *p* = 0.18). No biometric scale could be identified by LASSO models.

For the prediction of fatigue in the upcoming 12 months, no psychometric or biometric scale could be identified by LASSO models.

For the 1-year prediction of depressive mood out of all psychometric scales only “Social overload [*b* = 0.45 (95%CI -0.06, 0.96), *p* = 0.080)]and Over-commitment at work [*b* = 0.28 (95% CI -0.39, 0.95], *p* = 0.42] remained within the LASSO models.” No biometric scale could be identified by LASSO models.

### Most Predictive Pattern Set for 1-Year Prediction of Chronic Pain and Its Accuracy (Objective 3)

**FIGURE 4:**
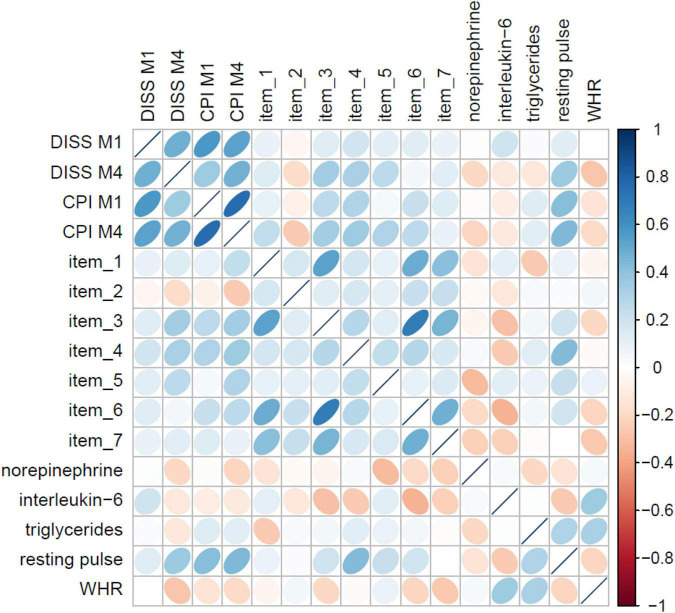
Correlation matrix of the seven psychometric items and five biometric markers from the LASSO models with pain M1 and M4.

The LASSO models on item level identified the most predictive (bio)marker set for non-specific CLBP consisting of seven psychometric items (out of 95 items) and five biometric markers (out of 30 markers), see Figure 4. Regarding the prediction accuracy for 1 year of these psychometric items, a RMSE = 12.63 for chronic pain intensity and RMSE = 9.81 for the subjective pain disability were found. The biomarker set reached a RMSE of 12.21 for CLBP intensity and a RMSE of 8.94 for CLBP disability, which meant that the prediction for pain disability only differed 8.94 points from the finally observed pain value 12 months later on a 0–100 point from von Korff scale; the prediction error was adjusted for age, gender, tobacco, alcohol, drug use, and physical activity.

**FIGURE 5:**
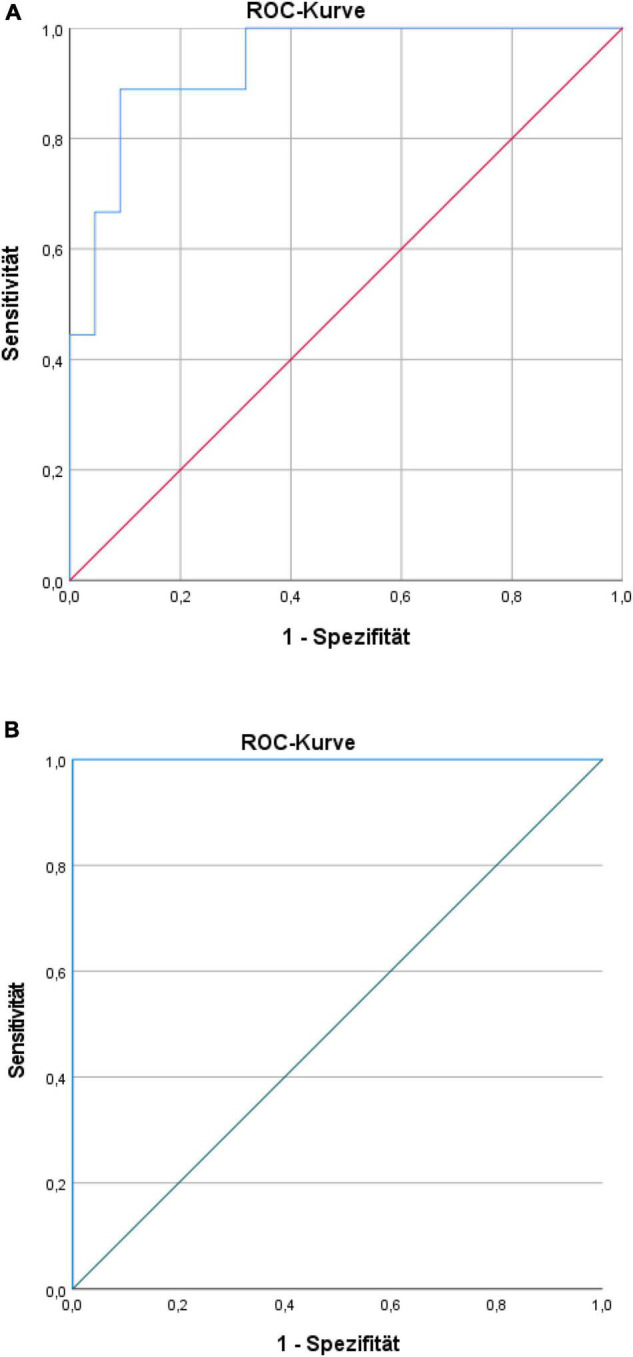
**(A)** Receiver-operating characteristic (ROC) curve for characteristic pain intensity prediction of the biomarker set. **(B)** ROC curve for pain disability prediction of the biomarker set.

Discriminant validity of the biomarker-set (ROC curve) showed an apparent AUC of 0.93 (95%CI: 0.85–1.00) for chronic pain intensity with a corrected AUC of 0.88 (bootstrapping by 728 iterations), the apparent AUC for subjective pain disability was 1.00 (95%CI: 1.00–1.00) with a corrected AUC of 0.99 (bootstrapping by 950 iterations) (see Figures 5A,B) [only in the case of subjective pain disability, our developing sample had very few participants with disability scores higher than 30 (*n* = 3)].

## Discussion

### Main Findings

First, the associations of stress with current LBP and the development on pain, fatigue, and depressive mood (individually as well as the appearance of a symptom triad) in chronic LBP (1-year after) were cross-sectionally and longitudinally evaluated through regression models. Associations of different types of stress (social, work-related, stress burden and critical life events) with current LBP or CLBP were observed. Furthermore, the same types of stress (excluding critical life events) together were associated with fatigue and mood in LBP (as symptom triad).

However, multiple inter-correlations between the stress types within the regression models were detected, indicating the need of statistical reduction procedures (such as LASSO).

Consequently, to be able to identify the most important stress type influencing the development of non-specific CLBP, fatigue, and depressive mood, a LASSO algorithm was applied. LASSO first results exhibited a reduction of influencing factors within a 1-year prediction; CLBP disability was best predicted by “Excessive demands at work,” while depressive mood was predicted by “Social overload” and “Over-commitment at work.” For fatigue, none of the stress types was selected.

Beyond and most relevant, to identify the most predictive stress-related (neuro)pattern set regarding the derivation of a diagnosis for the development of non-specific CLBP, LASSO models were further applied. Here, the results offered a psychometric-set based of seven simple questions and a biomarker set of five biomarkers (norepinephrine, interleukin-6, triglycerides, WHR, and resting pulse) with good predictive value.

### Comparison With Other Studies, Explanation of Findings

First results, similar to other studies, found that different types of stress (social, work-related, stress-burden, and critical life events) play a significant role in the appearance of current LBP and CLBP (46), fatigue, and depressive mood. Besides, a simultaneous presentation of pain, fatigue, and depressive mood as a symptom triad was observed. This symptom triad might be best explained by the overlapping neuroendocrinological mechanism between stress, pain, mood and fatigue (7, 8) and was in this study associated with almost all stress types at baseline.

A multimodal treatment strategy targeting physical and mental pain complaints—as it is often used in practice—make sense in light of these results. However, considering the influence of stress dimensions on CLBP after 1-year, results were less consistent. For example, on the one hand, the associations of chronic social, perceived or work-related stress and stress burden with current fatigue were observed, but only work-related stress and allostatic load (secondary ALI) seem to be relevant for the 1-year prediction. Similarly, current depressive mood was related to almost all stress types; but only work-related stress seemed to be relevant for the prediction of depressive mood within the upcoming year. Finally, the 12-months prediction of current pain was best described by the number of “critical life events” or “chronic worrying.” Therefore, although the data confirmed stress as a trigger (5, 6, 46) or an amplifier of pain (7, 8), methodological questions about prediction accuracy including multiple inter-correlations remained.

Ergo, the issue of a limited prediction quality by regression methods was addressed by applying a LASSO algorithm regarding the development of non-specific CLBP, fatigue, and depressive mood (second objective of this study). Here, all variables (scales) entered the LASSO model at the same time (as competitors), whereby only the strongest predictor remained in the model. In this way, variables were compared with each other while simultaneously controlling for baseline value, age, gender, and lifestyle factors (tobacco, alcohol consumption, sleep, physical activity, and income). Therefore, inter-correlations are prohibited.

Within the regression models, various different work-related stress scales were relevant for the 1-year prediction of fatigue and depression in CLBP, besides one social stress scale and life events for back pain. Otherwise in LASSO models, only two scales from work-related stress and one social stress type were relevant for the prediction of pain and depression in CLBP.

The last results and most important, intended to derive the “best-of” diagnostic set for CLBP (focus solely on pain) showed a psychometric-set based on seven questions and a biomarker set of five, with similar good predictive value. The psychometric diagnostic set reported a good accuracy in comparison to other psychometric stress screeners within the context of back pain [e.g., RPI-S domain stress with a RSME of 16.72 for CPI and of 16.20 for DISS (47)]. Nevertheless, at the moment there were already diagnostic stress screeners with appropriate sensitivity and specify values and cut-off for treatment personalization (45, 48–51); hence, the focus in this study was on the further development of the biomarker diagnostic set. The RMSE of the biomarker-set of only eight points already indicated a very good diagnostic accuracy with a very accurate clinical prediction sensitivity and specificity for pain intensity as well as disability (AUC 0.93 and 0.99, respectively); however, only few cases reported more than 30 points in the case of pain disability. The five biomarkers included in the set suggested that the prediction model for pain intensity might reside in parameters from the ergotropic system. The over-activity of the ergotropic system was generally associated with high noradrenergic activity, high blood pressure, cardiovascular output, and hypertension (52) as well as a reaction of the glandotropic system corresponding to mobilization of energy stores and metabolic processes. These processes fit to the neurochemical inflammatory mediator noradrenaline and interleukin-6, as well as with the metabolic parameter triglycerides, WHR and resting pulse identified in the study (all together grouped as “bio-set” consisting of the markers norepinephrine, interleukin-6, triglycerides, WHR and resting pulse).

A relation of stress and pain intensity is also conceivable for the reason that noradrenergic neurons have a variety of influences on pain inhibition (14), as well as on fat metabolism and fatty acid concentration (e.g., prostaglandins), which conversely influence the thickness of myelin sheaths and axon length; hence, axonal transport (53). The effect of local storage lipids on peripheral nerves functions is described elsewhere (16). Largely, the results may be best interpreted as meaning that chronic stress can be associated with a habituation of the glandotropic system (with blunted cortisol response, slight hypocortisolism and symptom triad), so that, for example, the prostaglandin synthesis (54) or an over-activation of the ergotropic system could not be sufficiently inhibited (55). Although the results can be understood in such a way and fit to the physiological pathways described in the introduction, many questions remain unanswered and should be investigated in a broader frame (56), for example, in further studies with considerably larger sample size.

### Strengths

The overall purpose of this study was to shed light on the question which stress types influence the development of non-specific CLBP. Considering that pain mostly appears in combination with fatigue and depressive symptoms these constructs were likewise included in the study. Further and most relevant, a diagnostic tool for practical use was introduced.

This longitudinal mixed-method study was conducted through a step-by-step methodologic procedure to approach the research questions in a structured way. Difficulties regarding different stress types simultaneously within multiple regression models (intercorrelations, see Figure 3) were addressed by specific selection models (e.g., LASSO), which allowed a selection of the most important predictor out of similar dimension within one model and the derivation of a diagnostic pattern set. This procedure highlighted the problems of translating theoretical knowledge into practical application, providing significant hints into understanding how stress influences the development of chronic pain disorders or the pain–fatigue–depression symptom triad and was a strength of this study.

Even though stress is often reported to be associated with pain becoming chronic (57), it becomes distinct in our results that the standard analytic models such as regression models and questionnaires alone might not be appropriate to deeply understand the underlying stress–pain pathways. Furthermore, regarding the stress-associated physiological scenarios on pain discussed at the beginning of this study, our data point out that a plain look at global biometric measures (ALI and HCC) may not be enough for an understanding and giving practical recommendations, even considering that mathematical algorithms were based and used across different biometric indices (58). Pain is represented in a network of biology, driven by genetic, cellular, neuronal, psycho-social or biomechanical triggers (59). For example, motor control exercise improves core stability, spine control, and muscle performance and is an important preventive or therapeutic strategy against CLBP (60, 61). On the other hand, neuromuscular adaption within the sensorimotor system is, for example, influenced by the neurotransmitter concentration. This reciprocal relationship between exercise, pain and stress (49) leads to limitations in the explanation of the pain network constellations (62). Therefore, the results in general may be conflicted or limited by methods or exclusion of important factors (e.g., physical activity/exercising, sleep) (59). Hence, in our LASSO model, physical activity was controlled for. Till today, it is only known that stress has an influence on pain becoming chronic; a remarkable finding from a twin study (63). However, less is known about developmental mechanisms and its detection at early stage for prevention (64). Indeed, our study recruited mostly young individuals to be able to follow them after an acute LBP episode, and so to derive a diagnostic tool with practical relevance.

### Limitations

(1) Due to the observational longitudinal design, the study did not include dynamic test procedures which might be better suited to evaluate the HPA-axis and make conclusions about hypo- or hyperactivity.

(2) Restricted sample size of the biomarkers assessments: It was difficult to recruit individuals who were willing to take part in the arduous physiological measurements (e.g., 12-h urine sampling overnight and nutrition protocol).

(3) We did not collect information about menstrual cycle phases of women. Indices such as ALI and HCC were less dependent of these phases, but other biomarkers could be, although there was no hormonal biomarker of relevance.

(4) Relatively small sample size for the applied selection procedure (LASSO), although by its mathematical construction LASSO was able to handle cases where the number of predictors was equal or greater than the sample size. However, the computational test sequences with decision trees (C4.5 algorithm selection) already came to similar results in the preliminary study, corresponding with first literature results from epidemiological data sets (65).

(5) Finally, ROC curves were only calculated in the developmental sample. An external validation of the screening by cross-validation in future studies was needed, and which was essential for the next developmental step.

(6) Development of simplified biomarkers measurements was desired. Practical application remains limited till now.

## Conclusion

The overall purpose of this study was to shed light on the question of how stress influences non-specific LBP and CLBP. It was shown that different stress types are associated with current LBP and CLBP, fatigue, and depressive mood. Work-related stress (e.g., Over-commitment, Excessive demands at work, and Effort), social stress (Chronic worrying and Social overload), and life events played role in the prediction of upcoming chronic pain complaints and depression in the next 12 months. Most importantly, it was possible to derive a “best-of” marker set for the prediction of a chronic course of LBP, once for psychometric items (7 items) and once for physiological markers (5 biomarkers). For the physiological marker set, additionally, a clinical prediction sensitivity and specificity was calculated with good accuracy. The psychometric items could be best sub-summarized under the broad concept of searching for sense of coherence in daily life, while the biomarker set gave information about possible mechanisms and physiological pathways between stress and pain, and the symptom triad. Finally, a step for step methodological procedure showed that very specific methods were needed to gain knowledge about these associations and their translation in clinical practice for an early screening of persons at risk for pain becoming chronic. One strength of this study was the presentation of the courses of different bio–psycho–social markers over 1 year in combination with lifestyle (such as sleep, physical activity, alcohol, tobacco, and medication) in regard to pain. Opening the possibility of formulating new research questions.

## Data Availability Statement

The raw data supporting the conclusions of this article will be made available by the authors, without undue reservation.

## Ethics Statement

The studies involving human participants were reviewed and approved by the Ethic Board University of Potsdam. The patients/participants provided their written informed consent to participate in this study.

## Author Contributions

P-MW: design, survey, first draft, and study Principal Investigator. DD and LPV: statistical analyses. P-MW, DD, and LPV: critical revision of the manuscript for important intellectual content. All authors contributed to the article and approved the submitted version.

## Conflict of Interest

The authors declare that the research was conducted in the absence of any commercial or financial relationships that could be construed as a potential conflict of interest.

## Publisher’s Note

All claims expressed in this article are solely those of the authors and do not necessarily represent those of their affiliated organizations, or those of the publisher, the editors and the reviewers. Any product that may be evaluated in this article, or claim that may be made by its manufacturer, is not guaranteed or endorsed by the publisher.

## Abbreviations

ALI: allostatic load index
AUC: area under the curve
BMI: body mass index
CLBP: chronic low back pain
CPI: chronic pain intensity
CUBP: chronic unspecific back pain
DHEA-S: serum dehydroepiandrosterone sulfate
DISS: pain disability
ECLIA: electrochemical luminescence immunoassay
EDTA: ethylendiaminetetraacetic acid
ECG: electrocardiogram
ELISA: enzyme-linked immunosorbent assay
ERI: effort–reward–imbalance questionnaire
GABA: gamma-aminobutyric acid
GPO–PAP: enzymatic color test
HADS-D: hospital anxiety and depression scale (German version)
HbA1c: glycated hemoglobin
HCC: hair cortisol concentration
HDL: high density lipoprotein
HPA: hypothalamus–pituitary–adrenal axis
HOMA: homeostasis model assessment
ICAM-1: intercellular adhesion molecule 1
ILE: inventory of life-changing events
LASSO: least absolute shrinkage and selection operator
LBP: low back pain
LDL: low density lipoprotein
M1 … M4: measuring points
*n*: number of participants
n.s.: not significant
POMS: profile of mood states, German short version
PSA3: parallel study 3
PSD10: parallel study 10
PSS: perceived stress scale
RMSE: root mean squared error
ROC: receiver-operating characteristic curves
TICS: Trier inventory for chronic stress
VAS: visual analog scale
VE: vital exhaustion
von Korff: chronic pain grade questionnaire
WHR: waist–hip ratio.
