# Supplementary material for: Stress and Pain. Predictive (Neuro)Pattern Identification for Chronic Back Pain: A Longitudinal Observational Study
Source: Front Med (Lausanne). 2022 May 10;9:828954. doi: 10.3389/fmed.2022.828954 (PMC9129900; doi:10.3389/fmed.2022.828954)

**Supplementary figure.** Allostatic load distribution (primary, secondary level and total) at baseline M1


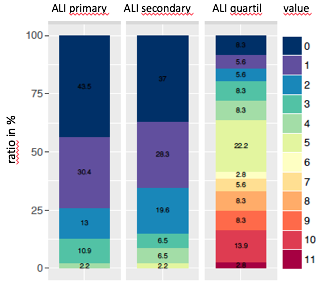

Supplement: Supplementary file 1 [file Data_Sheet_1.docx]
