# Supplementary material for: Stress and Pain. Predictive (Neuro)Pattern Identification for Chronic Back Pain: A Longitudinal Observational Study
Source: Front Med (Lausanne). 2022 May 10;9:828954. doi: 10.3389/fmed.2022.828954 (PMC9129900; doi:10.3389/fmed.2022.828954)
Supplement: Supplementary file 2 [file Table_1.docx]

**Supplementary Table 1.** Descriptive (M, SD) psychological and physiological measures at different time points (M1-M4).

|  |  |  | **M1** | | | **M2** | | | **M3** | | | **M4** | | |
| --- | --- | --- | --- | --- | --- | --- | --- | --- | --- | --- | --- | --- | --- | --- |
|  |  |  | ***n*** | ***M*** | ***SD*** | ***n*** | ***M*** | ***SD*** | ***n*** | ***M*** | ***SD*** | ***n*** | ***M*** | ***SD*** |
| **Outcome criteria**  **(pain-related measures)** | Chronic Pain  (CLBP) | CPI von Korff | 92 | 26.4 | 18.3 | 72 | 28.0 | 17.1 | 71 | 26.1 | 18.5 | 63 | 25.7 | 18.7 |
|  |  | DISS von Korff | 92 | 12.2 | 17.4 | 72 | 14.7 | 17.4 | 70 | 13.1 | 19.4 | 60 | 10.3 | 17.2 |
|  | Current Pain  (LBP) | VAS | 93 | 11.1 | 16.8 | 72 | 14.8 | 21.2 | 69 | 12.0 | 17.8 | 63 | 13.3 | 20.5 |
|  | Fatigue | Vital exhaustion | 91 | 8.1 | 4.3 | 71 | 8.5 | 4.5 | 71 | 7.0 | 5.0 | 63 | 8.5 | 4.9 |
|  |  | Fatigue POMS | 110 | 15.8 | 8.2 | 72 | 17.2 | 9.1 | 70 | 16.3 | 9.2 | 63 | 16.5 | 9.4 |
|  | Depressive mood | Depression POMS | 110 | 9.6 | 9.2 | 72 | 13.4 | 12.9 | 70 | 10.4 | 11.3 | 63 | 11.3 | 11.8 |
| **Stress types**  **(Psychometric questionnaires)** | Chronic Stress | Work Overload TICS | 92 | 15.1 | 6.8 | - | - | - | - | - | - | 63 | 13.9 | 6.4 |
|  |  | Social Overload TICS | 91 | 9.3 | 5.4 | - | - | - | - | - | - | 61 | 8.9 | 5.6 |
|  |  | Pressure to Perform TICS | 93 | 17.0 | 6.4 | - | - | - | - | - | - | 63 | 16.5 | 7.0 |
|  |  | Work Discontent TICS | 93 | 10.7 | 5.2 | - | - | - | - | - | - | 63 | 10.0 | 5.8 |
|  |  | Excessive Demands at Work TICS | 92 | 6.3 | 4.1 | - | - | - | - | - | - | 63 | 6.3 | 4.0 |
|  |  | Lack of Social Recognition TICS | 93 | 5.4 | 3.2 | - | - | - | - | - | - | 63 | 5.9 | 3.6 |
|  |  | Social Tensions TICS | 93 | 5.8 | 3.6 | - | - | - | - | - | - | 63 | 6.9 | 4.0 |
|  |  | Social Isolation TICS | 92 | 6.8 | 4.5 | - | - | - | - | - | - | 63 | 6.2 | 4.4 |
|  |  | Chronic Worrying TICS | 94 | 6.7 | 4.0 | - | - | - | - | - | - | 63 | 5.8 | 3.9 |
|  | Perceived Stress | Perceived Stress PSS | 94 | 18.0 | 6.6 | 94 | 18.0 | 6.5 | - | - | - | 61 | 16.6 | 7.0 |
|  | Stress at work (Effort-Reward-Imbalance) | Efford ERI | - | - | - | 71 | 8.1 | 2.2 | - | - | - | 63 | 7.7 | 2.2 |
|  |  | Reward ERI | - | - | - | 67 | 18.9 | 3.5 | - | - | - | 61 | 18.3 | 3.8 |
|  |  | Overcommitment ERI | - | - | - | 69 | 14.1 | 4.0 | - | - | - | 63 | 14.2 | 3.9 |
|  | Inventory of Life-Changing Events | Critical Live Events ILE | - | - | - | - | - | - | 71 | 8.5 | 6.2 |  | NA |  |
|  | Total Stress Index | Total Stress Index | 57 | 6.3 | 3.6 | - | - | - | - | - | - | 58 | 5.9 | 3.4 |
| **Biological measures**  **(Biometric assessments)** | Hair cortisol (HCC) | Hair Cortisol (pg/mg) | 110 | 20.0 | 17.5 | 73 | 19.6 | 15.1 | 76 | 19.0 | 13.4 | 60 | 88.0 | 389.1 |
|  | Sympathetic Nervous System (SNS) biomarkers | Epinephrine in urine (µg/d) | 46 | 7.0 | 4.3 | - | - | - | - | - | - | 36 | 3.7 | 2.9 |
|  |  | Norepinephrine in urine (µg/d) | 46 | 29.6 | 21.5 | - | - | - | - | - | - | 36 | 14.9 | 7.5 |
|  | Parasympathetic Nervous System (PNS) biomarkers | SDNN Heart rate variability indicator | 42 | 70.9 | 31.5 | - | - | - | - | - | - | 36 | 71.6 | 31.6 |
|  |  | RMSSD Heart rate variability indicator | 42 | 45.1 | 30.3 | - | - | - | - | - | - | 36 | 42.2 | 20.0 |
|  |  | SDANN Heart rate variability indicator | 42 | 23.2 | 16.3 | - | - | - | - | - | - | 35 | 19.3 | 19.6 |
|  |  | SDNNidx Heart rate variability indicator | 42 | 65.8 | 28.7 | - | - | - | - | - | - | 36 | 67.9 | 29.0 |
|  | Hypothalamic Pituitary Adrenal axis (HPA-Axis) | Cortisol in urine (µg/d) | 46 | 125.3 | 56.6 | - | - | - | - | - | - | 36 | 126.6 | 51.2 |
|  |  | Serum DHEA-S (µg/d) | 46 | 0.7 | 0.7 | - | - | - | - | - | - | 36 | 0.9 | 0.6 |
|  | Immune System biomarkers | Serum Interleukin-6 (IL-6) (pg/ml) | 46 | 0.6 | 1.6 | - | - | - | - | - | - | 36 | 1.2 | 1.5 |
|  |  | Fibrinogen (mg/dL) | 46 | 282.4 | 56.8 | - | - | - | - | - | - | 36 | 261.3 | 40.7 |
|  |  | C- Reactive Protein (mg/dL) | 46 | 1.0 | 1.7 | - | - | - | - | - | - | 36 | 1.2 | 1.9 |
|  |  | E-Selectin (ng/ml) | 46 | 31.6 | 17.8 | - | - | - | - | - | - | 36 | 36.7 | 16.5 |
|  |  | Serum ICAM-1 (ng/ml) | 46 | 283.3 | 98.8 | - | - | - | - | - | - | 36 | 265.2 | 51.4 |
|  | Cardiovascular biomarkers | Systolic Blood Pressure (mmHg) | 45 | 103.0 | 11.2 | - | - | - | - | - | - | 36 | 108.2 | 14.5 |
|  |  | Diastolic Blood Pressure (mmHg) | 45 | 66.4 | 8.9 | - | - | - | - | - | - | 36 | 69.6 | 9.1 |
|  |  | Resting pulse (bpm) | 42 | 67.9 | 12.9 | - | - | - | - | - | - | 36 | 65.0 | 11.0 |
|  | Lipid Metabolic Biomarkers | Body mass index (BMI) (kg/m^2^) | 45 | 23.4 | 3.5 | - | - | - | - | - | - | 36 | 23.8 | 3.8 |
|  |  | Waist-to-Hip-Ratio (WHR) | 45 | 0.8 | 0.1 | - | - | - | - | - | - | 36 | 0.8 | 0.1 |
|  |  | Serum Triglycerides (mg/dL) | 46 | 91.1 | 31.2 | - | - | - | - | - | - | 36 | 92.1 | 41.7 |
|  |  | Serum HDL Cholesterol (mg/dL) | 46 | 63.8 | 12.7 | - | - | - | - | - | - | 36 | 64.9 | 14.2 |
|  |  | Serum LDL Cholesterol (mg/dL) | 46 | 113.4 | 29.4 | - | - | - | - | - | - | 36 | 113.4 | 29.2 |
|  | Glucose Metabolic Biomarkers | Glycosylated hemoglobin (HbA1c) (%) | 46 | 5.0 | 0.4 | - | - | - | - | - | - | 36 | 0.1 | 0.1 |
|  |  | Fasting glucose (mg/dL) | 46 | 87.0 | 6.1 | - | - | - | - | - | - | 36 | 86.5 | 6.2 |
|  |  | Fasting insulin (µU/ml) | 45 | 6.0 | 3.5 | - | - | - | - | - | - | 36 | 6.6 | 7.9 |
|  | Allostatic Load Index | Primary allostatic load | 46 | 1.0 | 1.1 | - | - | - | - | - | - | 36 | 1.0 | 1.1 |
|  |  | Secondary allostatic load | 46 | 1.3 | 1.5 | - | - | - | - | - | - | 36 | 1.5 | 1.3 |
|  |  | Total allostatic load (ALI quartil, 24 biomarkers) | 41 | 5.4 | 3.3 | - | - | - | - | - | - | 35 | 5.9 | 2.8 |
|  | M: Mean; SD: Standard deviation; NA: Do not apply | | | | | | | | | | | | | |
